# Successful Treatment of Obstructive Ureterolithiasis with Medical Expulsive Therapy Including Tamsulosin in a Dog

**DOI:** 10.3390/vetsci13010069

**Published:** 2026-01-10

**Authors:** Chaeyeon Park, Yelim Lee, Yeon Chae, Taesik Yun, Byeong-Teck Kang, Hakhyun Kim

**Affiliations:** Laboratory of Veterinary Internal Medicine, College of Veterinary Medicine, Chungbuk National University, Cheongju 28644, Chungbuk, Republic of Korea; ac5500@naver.com (C.P.); lyr824@naver.com (Y.L.); bluesfiddle@naver.com (Y.C.); fermium@chungbuk.ac.kr (T.Y.); kangbt@chungbuk.ac.kr (B.-T.K.)

**Keywords:** canine, ureteral obstruction, stone expulsion, medical expulsive therapy, alpha-adrenergic antagonist, tamsulosin

## Abstract

Ureterolithiasis is a common cause of ureteral obstruction in dogs, often leading to kidney injury. Medical expulsive therapy (MET) using α-adrenergic antagonists has been proposed as a nonsurgical treatment option in selected cases and is thought to facilitate ureteral stone passage by reducing ureteral smooth muscle tone. A 9-year-old castrated male Chihuahua weighing 1.78 kg was presented with anorexia. Physical examination revealed 7% dehydration and pale mucous membranes. Serum biochemistry demonstrated severe azotemia, with markedly elevated symmetric dimethylarginine (>100 μg/dL; reference interval [RI], 0–14 μg/dL), blood urea nitrogen (157.9 mg/dL; RI, 7–25 mg/dL), and creatinine (2.2 mg/dL; RI, 0.5–1.5 mg/dL). On day 4 of hospitalization, ultrasonography revealed dilation of the renal pelvis (16.1 mm), ureteral distention (3.74 mm), and multiple ureteroliths (maximum diameter, 3.31 mm) at the ureterovesical junction. Antegrade pyelography confirmed a right ureteral obstruction. As the owner declined surgical intervention, MET including tamsulosin, was initiated with close clinical monitoring. After 3 days, improvement in azotemia and resolution of ureteral obstruction were observed. Although concurrent medical treatments were administered, this case provides clinical insight into the potential role of tamsulosin as part of medical management of obstructive ureterolithiasis in a dog with small distal ureteral stones.

## Simple Summary

Ureterolithiasis is a common cause of urinary obstruction in dogs and can lead to kidney injury if not treated promptly. Although surgery is usually the treatment of choice, it may not always be possible because of anesthesia risks or financial limitations. In some cases, medical expulsive therapy (MET), such as α-adrenergic antagonists, can help facilitate the passage of ureteroliths. This case report describes a 9-year-old Chihuahua with anorexia, dehydration, and kidney dysfunction caused by obstructive ureteroliths (maximum diameter, 3.31 mm) in the distal ureter. Because surgery was not elected by the owner, the dog was treated with tamsulosin, an α-adrenergic antagonist that relaxes the ureter and facilitates the expulsion of small distal ureteroliths. After treatment, the stones passed within 72 h, and kidney function improved significantly. This report suggests that tamsulosin may be considered a minimally invasive treatment option for obstructive ureterolithiasis in selected dogs for whom surgery is declined due to financial constraints or perceived risk.

## 1. Introduction

Ureteral obstruction, which can result in severe pain and significant kidney damage, necessitates prompt intervention, especially when the blockage is severe [1]. The common causes of ureteral obstruction in dogs include ureterolithiasis, trigonal neoplasia, ureteral strictures, and blood clots, with ureterolithiasis being the most prevalent [2].

Treatment options for ureterolithiasis include medical expulsive therapy (MET), shock wave lithotripsy, and surgeries such as ureteral stenting, subcutaneous ureteral bypass (SUB), ureteral incision and ureteroneocystostomy [3]. Surgical interventions, although frequently recommended, are associated with risks including anesthesia complications, infection, re-obstruction, and high costs. MET, including fluid therapy, diuretics, calcium channel blockers, and α-adrenergic antagonists, is a non-invasive alternative considered for ureterolithiasis under 4 to 5 mm in size and not associated with infection, within the first 24–72 h [3,4].

Sympathetic innervation plays a crucial role in ureteral contraction, with alpha-1 adrenergic receptors distributed in ureteral smooth muscle [3]. Alpha-1 adrenergic antagonists, such as tamsulosin, induce ureteral relaxation, thereby facilitating the passage of urinary calculi in humans [5].

In human medicine, tamsulosin is commonly prescribed for ureteral stones, although reported clinical outcomes have been variable [6,7,8,9]. In cats, clinical use of tamsulosin for obstructive ureterolithiasis has also been reported; however, the available evidence remains limited and is primarily derived from small case series [10].

Reported adverse effects of tamsulosin in humans include hypotension, dizziness, and mild gastrointestinal signs [7,8]. In cats treated with tamsulosin for obstructive ureteral stones, clinically relevant adverse effects have been rarely reported [10]. In dogs, however, the efficacy and safety of tamsulosin for ureteral obstruction have not been well established. This case report describes successful expulsion of obstructive ureteroliths in a dog treated with MET including tamsulosin.

## 2. Case Presentation

**Figure 1:**
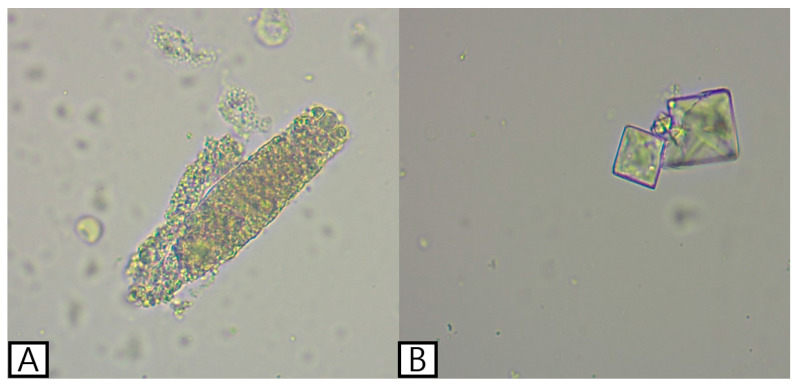
Urine sediment examination showing a granular cast (**A**) and calcium oxalate crystals (**B**) in the present case.

A 9-year-old castrated male Chihuahua weighing 1.78 kg was referred for panting and anorexia. Physical examination revealed tachypnea (90 breaths/min), 7% dehydration, pale mucosal membranes, and a systolic arterial blood pressure (SBP) of 142 mmHg. Complete blood cell count (CBC) revealed leukocytosis (34.47 × 10^3^/μL; reference interval [RI], 5.05–16.76 × 10^3^/μL) with neutrophilia (31.63 × 10^3^/μL; RI, 2.95–11.64 × 10^3^/μL). Packed cell volume was mildly decreased (31.6%; RI, 37.3–61.7%). Serum biochemical analysis included increased symmetric dimethylarginine (SDMA) (>100 μg/dL; RI, 0–14 μg/dL), blood urea nitrogen (BUN) (157.9 mg/dL; RI, 7–25 mg/dL), creatinine (2.2 mg/dL; RI, 0.5–1.5 mg/dL) and C-reactive protein (CRP) (322.15 mg/L; RI, 0–10 mg/L). Electrolyte imbalances included hyponatremia (136 mmol/L; RI, 141–152 mmol/L), hypochloremia (101 mmol/L; RI, 105–115 mmol/L), hypocalcemia (8.0 mg/dL; RI, 9–11.3 mg/dL), and hyperphosphatemia (9.8 mg/dL; RI, 2.6–6.2 mg/dL). Coagulation testing revealed elevated D-dimer (778.96 ng/mL; RI, 0–250 ng/mL). Urinalysis revealed a urine specific gravity of 1.015 (RI, 1.015–1.045), an elevated urine protein-to-creatinine ratio (UPC) (2.79; RI, 0–0.5), a urine pH of 5.0, granular casts, calcium oxalate crystals, and negative bacterial cultures (Figure 1).

**Figure 2:**
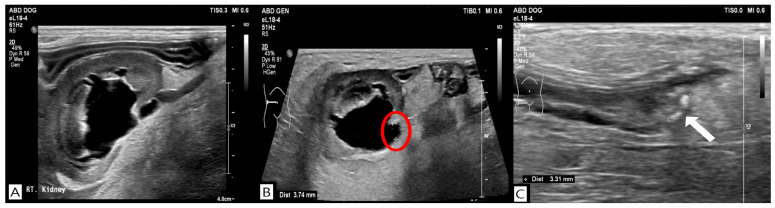
Ultrasonographic findings on day 4 revealed severe dilation of the right renal pelvis (**A**), distention of the right ureter (red circle) (**B**), and the presence of ureteral calculi (white arrow) (**C**).

No remarkable abnormalities were observed on thoracic radiography. Abdominal radiography performed in the right lateral and ventrodorsal projections showed decreased serosal detail, gastric gas dilation, and an irregular margin in the left kidney, whereas the right kidney was poorly visualized. No radiopaque uroliths were identified on radiography. Abdominal ultrasonography was performed using an ultrasound system (Philips EPIQ 7^®^, Philips Healthcare, Andover, MA, USA) equipped with a linear array transducer (EL18-4, Philips Healthcare, Andover, MA, USA; 2–22 MHz), revealing a hyperechoic peritoneum and irregular kidney margins. Notably, the right kidney exhibited marked dilation of the renal pelvis (14.2 mm) and proximal ureteral dilation (maximum, 3 mm) without evidence of calculi. Differential diagnoses for hydronephrosis included pyelonephritis, ureteral stenosis, ureteral obstruction, and an ectopic ureter. Pyelonephritis was considered the most likely condition in the absence of identifiable calculi. Initial treatment included empirical antibiotic therapy with ampicillin-sulbactam (Sulbacin^®^, Shinpoong Pharmaceutical Co., Seoul, Republic of Korea; 20 mg/kg, intravenously, three times daily) and enrofloxacin (Baytril^®^, Elanco Animal Health Incorporated, Greenfield, IN, USA; 3 mg/kg, subcutaneously, twice daily), analgesia with lidocaine (Daihan Lidocaine HCL Hydrate^®^, Daehan Pharmaceutical Co., Seoul, Republic of Korea; 1 mg/kg/h)—ketamine (Hanall Ketamine HCL^®^, HanAll Biopharma Co., Seoul, Republic of Korea; 0.2 mg/kg/h) continuous rate infusion. Because the D-dimer level was markedly elevated, dalteparin (Fragmin^®^, Pfizer, Puurs, Belgium; 150 IU /kg, subcutaneously, three times daily) was administered prophylactically to reduce thromboembolic risk. Fluid therapy with lactated Ringer’s solution (Hartmann’s solution^®^, JW Pharmaceutical, Gwacheon, Republic of Korea) was administered to correct 7% dehydration for 6 h. Once hydration improved and the target body weight was achieved, the infusion rate was reduced to the maintenance fluid rate, which was continued until discharge. On day 4 of hospitalization, ultrasonography showed worsening dilation of the right renal pelvis (16.1 mm) and ureteral dilation (3.74 mm), with distal ureteroliths (maximum diameter, 3.31 mm), as shown in Figure 2.

**Figure 3:**
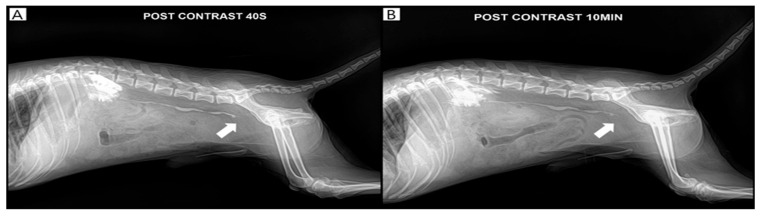
Pyelography confirmed the right ureteral obstruction (white arrow). Contrast agent retention of the kidney and ureter was observed after 10 min of contrast on day 4. (**A**): 40 s post-contrast pyelography, (**B**): 10 min post-contrast pyelography.

Antegrade pyelography was performed following percutaneous ultrasound-guided pyelocentesis under butorphanol sedation (Butophan Inj.^®^, Myungmoon Pharm. Co., Seoul, Republic of Korea; 0.2 mg/kg, intravenously), with aseptic skin preparation, to evaluate for other potential causes of ureteral obstruction, such as an ectopic ureter or ureteral stricture. Using a 23-gauge needle, 2 mL of urine was aspirated from the renal pelvis, followed by antegrade intrapelvic injection of 1 mL of iohexol (300 mg iodine/mL; Omnipaque^®^ 300, GE Healthcare Co., Ltd., Shanghai, China) [11]. Abdominal radiographs were subsequently obtained after patient repositioning. Persistent contrast retention at 10 min confirmed ureteral obstruction (Figure 3).

**Figure 4:**
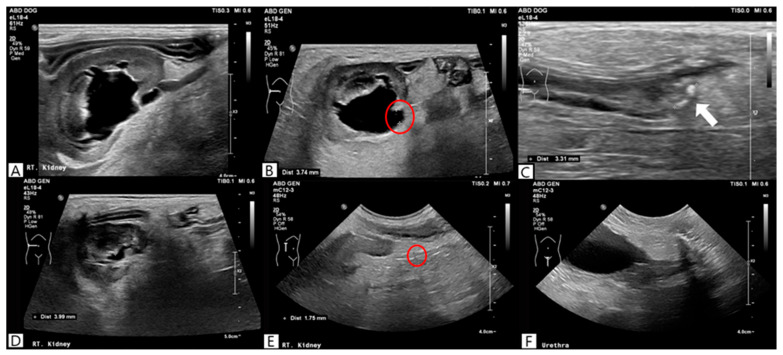
Ultrasonographic findings before (**A**–**C**) and after medical expulsive therapy. Post-treatment ultrasonography on day 7 demonstrated a reduction in right renal pelvic dilation (**D**) and ureteral distention (red circle) (**E**), with no detectable ureteroliths (**F**).

Bacterial culture of the renal pelvic fluid obtained during the procedure was negative results. However, because the sample was collected after the initiation of antimicrobial therapy, pyelonephritis could not be definitively excluded. Therefore, antimicrobial therapy was continued during hospitalization in consideration of leukocytosis, increased CRP concentration, and the high incidence of concurrent urinary tract infection [4]. Due to the financial constraints for surgery, medical expulsive therapy with tamsulosin (Hanmi Tams cap.^®^, Hanmi Pharmaceutical Co., Seoul, Republic of Korea; 0.1 mg/kg, per os [PO], once daily) was initiated. Because tamsulosin has been associated with adverse effects such as hypotension, dizziness, and gastrointestinal signs, the patient was closely monitored during treatment. SBP was measured using an oscillometric blood pressure monitoring device (Cardell^®^ Insight Diagnostic Monitor, Midmark Corporation, Versailles, OH, USA). An appropriately sized cuff (SV2) was applied to the right forelimb and positioned as close to heart level as possible. Blood pressure measurements were obtained while the dog was seated and remained motionless. Prior to tamsulosin administration, the dog consistently maintained SBP values of ≥120 mmHg. Following initiation of tamsulosin therapy, SBP decreased to 97 mmHg at 12 h. Based on this reduction in SBP, although no overt clinical signs suggestive of hypotension were observed, the tamsulosin dose was reduced to 0.05 mg/kg once daily and subsequently maintained. Based on evidence that MET with tamsulosin combined with furosemide could be an effective for the treatment distal ureter stones in humans [12], furosemide (Laxis^®^, Handok Pharmaceuticals Co., Eumseong, Republic of Korea; 0.7 mg/kg, intravenously, three times daily) was added as adjunctive therapy to maintain the effectiveness of MET following tamsulosin dose reduction. No further episodes of hypotension were observed during furosemide administration. Follow-up ultrasonography confirmed spontaneous stone passage 3 days after initiation of tamsulosin therapy, with right renal pelvic dilation reduced to 3.99 mm and ureteral diameter reduced to 1.75 mm (Figure 4).

Blood parameters also significantly improved, including SDMA (60 μg/dL), BUN (44.0 mg/dL), creatinine (1.9 mg/dL), and CRP (136.9 mg/L). Therefore, tamsulosin and furosemide therapy were discontinued. The dog remained hospitalized because of concurrent pancreatitis. Follow-up ultrasonography was performed on days 8, 9, and 11, and no recurrence of renal pelvic dilation, ureteral obstruction, or ureterolithiasis was identified. The patient was discharged on day 12 of hospitalization.

## 3. Discussion

This case report describes a dog with obstructive ureterolithiasis that was managed nonsurgically using tamsulosin as part of the MET. Although tamsulosin has been studied for managing ureterolithiasis in humans and cats, studies evaluating its efficacy in dogs are limited [3,7,8,10]. This case provides clinical insights into the potential use of tamsulosin as a non-surgical therapeutic option for managing ureteral obstruction caused by ureteroliths in dogs.

The dog had right distal ureteral obstruction with azotemia and unilateral hydronephrosis requiring immediate intervention [4]. In dogs, approximately 50–60% of ureteroliths are calcium-based and considered insoluble [4,13]. Even for potentially dissolvable struvite stones, medical or dietary dissolution is not advised after obstruction occurs because of the risk of kidney injury [3,13]. Surgery was initially considered but was declined for financial reasons. In this case, the ureteroliths were over 3 mm in diameter. This size is above the reported natural passage threshold of 2.8 mm in beagles [3]. Therefore, spontaneous excretion was unlikely. Despite the initiation of fluid therapy on the first day of hospitalization, no passage of ureteroliths was observed, indicating that the infusion rate alone was insufficient to facilitate expulsion. Consequently, tamsulosin was initiated as part of the MET. Several human studies have reported that ureterolithiasis in patients with calculi less than 5 mm located in the distal ureter can be treated nonsurgically with alpha-adrenergic antagonists [8,14]. The maximum ureteral diameter in dogs is approximately 2.7 mm, which is less than the average human ureteral diameter of 3 mm [3,15]. Therefore, findings from human studies may inform clinical expectations for the passage of ureteroliths of approximately 3 mm in diameter in dogs. A recent study examined 70 cases of obstructive ureterolithiasis in cats. Tamsulosin alone led to successful stone passage in 31.4% of patients [10]. This observation indicates that the pharmacological mechanism by which tamsulosin facilitates ureteral stone expulsion in humans and cats may also be relevant to dogs. However, despite these observations, the overall evidence supporting the efficacy of tamsulosin remains limited and inconclusive. Although tamsulosin is widely used as MET in human patients with ureterolithiasis, clinical outcomes have been inconsistent across studies, and its overall efficacy remains controversial [6,9]. Similarly, in veterinary medicine, the use of tamsulosin for obstructive ureterolithiasis is off-label and the available evidence in cats is limited to small case series, whereas data in dogs remain lacking [10].

Transient hypotension developed during treatment, but resolved after dose reduction and supportive care. This adverse effect is consistent with previous studies suggesting that tamsulosin’s affinity for alpha-1B adrenoceptors in vascular smooth muscle may transiently reduce systemic blood pressure [16]. Accordingly, tamsulosin should be used with caution in dogs with severe cardiac disease, pre-existing hypotension, or hemodynamic instability; therefore, careful monitoring and individualized dosing are essential. In those cases, the use of lower dosages of tamsulosin (e.g., 0.05 mg/kg) could represent a more appropriate and safer therapeutic approach.

This case report has some limitations. One limitation of this case is that distal ureteroliths were not identified on ultrasonography at the time of presentation. Both stone migration and limited detectability during the initial examination should be considered. Visualization of the distal ureter on ultrasonography can be technically challenging, particularly under suboptimal imaging conditions, and may be influenced by operator experience. Accordingly, the distal ureteroliths could be already present at presentation but were not detected on the initial examination. Secondly, the potential synergistic effect of furosemide should be considered. Furosemide was administered on day 5 and may have influenced ureteral stone passage. Its diuretic effect increases urine flow and ureteral peristalsis [12]. Therefore, this case may support a combined therapeutic approach of ureterolithiasis, in which furosemide and tamsulosin act synergistically in dogs. Although spontaneous ureteral stone passage was considered unlikely based on stone size, inter-individual variability cannot be excluded when interpreting the clinical response.

## 4. Conclusions

This case report describes the use of MET including tamsulosin in a dog with obstructive ureterolithiasis. Tamsulosin may be considered as a medical management option for ureterolithiasis in dogs with small distal ureteral stones. In addition, furosemide might be considered as an adjunctive therapy when used in combination with tamsulosin in dogs with ureterolithiasis. Further prospective and controlled studies are required to confirm the efficacy of tamsulosin, determine optimal dosing regimens, and assess its safety in the medical management of ureterolithiasis in dogs.

## Data Availability

The data presented in this study are available on request from the corresponding author because data sharing requires the consent of the authors.

## Abbreviations

The following abbreviations are used in this manuscript:

MET	medical expulsive therapy
SDMA	symmetric dimethylarginine
RI	reference interval
BUN	blood urea nitrogen
CRP	C-reactive protein
UPC	urine protein-to-creatinine ratio
PO	per os
